# Insecticidal Activity and Chemical Composition of the *Morinda lucida* Essential Oil against Pulse Beetle *Callosobruchus maculatus*

**DOI:** 10.1155/2014/784613

**Published:** 2014-07-20

**Authors:** Moses S. Owolabi, Eduardo Padilla-Camberos, Akintayo L. Ogundajo, Isiaka A. Ogunwande, Guido Flamini, Olaniyi K. Yusuff, Kirk Allen, Karen Isabel Flores-Fernandez, Jose Miguel Flores-Fernandez

**Affiliations:** ^1^Department of Chemistry, Lagos State University, PMB 0001, Lasu Post Office, Ojo, Lagos 102001, Nigeria; ^2^Biotecnología Médica y Farmacéutica, Centro de Investigación y Asistencia en Tecnología y Diseño del Estado de Jalisco, AC, Avenida Normalistas 800, Col. Colinas de la Normal, 44270 Guadalajara, JAL, Mexico; ^3^Dipartimento di Chimica Bioorganica e Biofarmacia, Universita di Pisa, Via Bonanno 33, 56126 Pisa, Italy; ^4^Ingeniería en Biotecnología, Universidad Politécnica del Valle de Toluca, Carretera Toluca-Almoloya de Juárez km 5.6, Camino la Loma, Santiaguito Tlalcilalcali, 50900 Almoloya de Juárez, Mexico

## Abstract

Insecticidal activity of essential oil extracted from *Morinda lucida* was tested on pulse beetle *Callosobruchus maculatus*, which is a pest that causes serious damage to several pulses. The insecticidal activity was compared with two pesticides, Phostoxin and Primo-ban-20. 120 mixed sex adult *C. maculatus* were introduced, along with 30 g of cowpeas. Four concentrations (0.40, 0.20, 0.10, and 0.05 *μ*g/mL) of the *M. lucida* essential oil, Phostoxin, and Primo-ban-20 were tested. Essential oil chemical composition was analyzed by GC-MS. *M. lucida* essential oil showed a high toxicological effect, producing 100% mortality after 72 hours at a dose of 0.20 *μ*g/mL. *M. lucida* essential oil had a potent insecticidal activity (LC_90_ = 0.629 *μ*g/mL) compared to both pesticides, Phostoxin (LC_90_ = 0.652 *μ*g/mL) and Primo-ban-20 (LC_90_ = 0.726 *μ*g/mL), at 24 h. The main compounds of the essential oil were the oxygenated monoterpenoids, 1,8-cineole (43.4%), and *α*-terpinyl acetate (14.5%), and the monoterpene hydrocarbons, mostly sabinene (8.2%) and *β*-pinene (4.0%). Results clearly indicate that *M. lucida* essential oil can be used as an effective alternative for pulse beetle *C. maculatus* control, and it could be tested against other pulse beetles affecting Asia and Africa and throughout the world, thereby reducing use of synthetic pesticides.

## 1. Introduction

*Callosobruchus maculatus *is an economically important pest of several pulses, including leguminous grains such as lentils, cowpeas, green gram, chickpea, black gram, soybean, and haricot beans [1, 2]. These pulses are important sources of vegetable protein for millions of people of tropical and subtropical regions of Asia and Africa.* C. maculatus *larvae bore into pulse grain or into stored crop, making it unfit for human consumption and unviable for replanting [3–5].

Control of insect pestsrelies heavily on the use of synthetic insecticides such as methyl bromide or phosphine. However, their intensive use has led to the development of genetic resistance by insect species, pest resurgence, residual toxicity, environmental hazards, and serious problems arising from factors such as direct toxicity to predators, pollinators, fish, and man [6]. As such, the use of methyl bromide is being restricted because of its potential to damage the ozone layer [7]. Susceptibility of crop plants to insect pests and increasing costs of application of the presently used synthetic pesticides have directed the need for effective, biodegradable pesticides [8, 9].

Because of their high volatility, plant extracts or plant compounds and the use of natural compounds with insecticidal activity provide a potential biodegradable alternative to synthetic pesticides [10–12]. The contact and fumigant insecticidal activity of several plant essential oils and their constituents have been demonstrated against stored-product pests [13–15], a number of bruchid pests [16–18], and insects and mites [19].

Several botanical families for pest control are described, such as Meliaceae, Rutaceae, Asteraceae, Labiatae, and Malvaceae. Rubiaceae is a family of 630 genera and about 13,000 species [20].* Morinda lucida *(Benth), a member of Rubiaceaefamily, is a medicinal plant of about 15 m tall with a dense crown of slender crooked branches approximately 20–30 cm in diameter.* M. lucida *commonly known by the Yoruba in southwestern part of Nigeria as “O*ruwo*” is widely distributed in West Africa and is used in African folk medicine to treat several diseases [21]. The leaves are bitter and are used by the natives to treat malaria, yellow fever, jaundice, hepatitis, eczema, edema, cough, hypertension, diabetes, and sexual weakness [20, 22, 23].

In a previous study, another species of the same genus,* M. citrifolia,* showed larvicidal activity against three species of mosquito vectors [24] and the biofriendly nature of* M. lucida* suggested the need to search for new properties of this plant; therefore the present study was conducted to determine the insecticidal activity of the essential oil of* M. lucida* against* C. maculatus* by dose response bioassays and to characterize its chemical composition for consideration as an alternative insecticide for pulse beetle.

## 2. Materials and Methods 

### 2.1. Plant Material

The aerial parts of the* M. lucida *were collected from Apomu (7°19′48N, 4°10′47E), which is located in Osun State, Nigeria, and identified at the Herbarium of the Botany Department, University of Lagos, Nigeria, where voucher specimen number LUH 2637 was kept. The samples were air-dried, pulverized, and stored in polythene bag to reduce evaporation of the volatile oil.

To extract the essential oil, 500 g of pulverized sample was placed in a 5 L flask and distilled water was added to cover sample. Essential oil was obtained by hydrodistillation using glass modified Clevenger-type apparatus; the process ran for 4 h at normal atmospheric pressure and at 96-97°C inside the extractor. After the essential oil isolation, residual water was removed by filtration with anhydrous sodium sulphate, and then the essential oil was stored in an amber vial at 4°C for future analysis.

### 2.2. Gas Chromatography (GC) Analysis

GC analysis was performed with a HP-5890 Series II instrument equipped with a HP-Wax and HP-5 capillary columns (both 30 m × 0.25 mm ID × 0.25 *μ*m film thickness), working with the following temperature program: 60°C for 10 min, rising at 5°C/min to 220°C. The injector and detector temperatures were maintained at 250°C; carrier gas nitrogen (2 mL/min); detector dual, FID; split ratio 1 : 30. The volume injected was 0.5 *μ*L. The relative proportions of the oil constituents were percentages obtained by FID peak-area normalization without the use of response factor.

### 2.3. Gas Chromatography-Mass Spectrometry (GC-MS)

GC-MS analysis was performed with a Varian CP-3800 gas chromatograph equipped with a HP-5 capillary column (30 m × 0.25 mm ID × 0.25 *μ*m film thickness) and a Varian Saturn 2000 ion trap mass detector. Analytical conditions were injector and transfer line temperature 220°C and 240°C, respectively; oven temperature was programmed from 60 to 240°C at 3°C/min. Carrier gas was helium at 1.0 mL/min flow rate. Injection volume was 0.2 *μ*L (10% hexane solution) using a 1 : 30 split ratio. The mass spectrometer was operated at 70-eV ionization voltage. The acquisition mass range was 30 to 300 *m*/*z* at 1.0 scan/s.

### 2.4. Identification of the Volatile Compounds

The identification of the volatile constituents of the essential oil sample was made using the following criteria: (1) comparison of mass spectra with the Wiley library spectra 275 L (Rev.C.00.00) electronic database; (2) injection of authentic compounds from Sigma-Aldrich with a 95% minimum purity under the same analytical conditions; and (3) comparison of the retention index (RI) obtained by GC-MS with the RI theoretical in similar phases. The molecular weights of all the identified substances were confirmed by GC-MS, using MeOH as CI ionizing gas.

### 2.5. Insect Culture

*Callosobruchus maculatus *were reared on cowpea seeds (*Vigna unguiculata*) under controlled temperature and humidity at 28 ± 1°C, 58 ± 5% RH (12 h light cycle) and 25.5 ± 1°C, 45 ± 5% RH (12 h dark cycle). Adult insects of 8 days old were used for insecticidal test.

All experiments were carried out under the same environmental conditions as described above.

### 2.6. Insecticidal Activity of* M. lucida *Essential Oil

The insecticidal activity was evaluated as described by Ilboudo et al. [25]. One hundred and twenty mixed sex adult* C. maculatus* (8 days old) were put into a 500 mL glass bottle with 30 g of cowpea seeds and kept in the laboratory at 28 ± 1°C, 58 ± 5% RH (12 h light cycle) and 25.5 ± 1°C, 45 ± 5% RH (12 h dark cycle) for 72 h. Concentrations of the* M. lucida *essential oil diluted in acetone were tested on* C. maculatus* (0.05, 0.10, 0.20, and 0.40 *μ*g/mL). The appropriate concentrations were applied to filter paper (Whatman number 1, cut into 7 cm diameter) and immediately introduced into a glass bottle that was then hermetically sealed. For the control group, the insects were placed in the glass bottles under the same conditions but without the essential oil. Each concentration and control was replicated three times. Insect mortality was determined by observing the recovery of immobilized insects in 12 h intervals up to 72 h. When no antennal or leg movements were observed, insects were considered dead.

### 2.7. Insecticidal Activity of Commercial Pesticides

Two synthetic pesticides were purchased from Chemical and Allied company, Lagos, Nigeria. Phostoxin (55% Aluminium phosphide) and Primo-ban-20 (pirimiphos-methyl, emulsifiable concentrate 50%). The first is converted to phosphine gas, which is taken by the insect through its respiratory system and the second is able to penetrate the insect cuticle. Pellets of Phostoxin were divided into small pieces and weighed to achieve the appropriate concentrations and were applied according to manufacturer's directions [26]. Primo-ban-20 was diluted to achieve the desired concentrations, from a stock solution of 2 *μ*g/mL. Both pesticides were evaluated at the same concentrations as the essential oil in triplicate.

### 2.8. Mortality Determination

Ten pairs of adult* C. maculatus* were released each 12 h after exposure of essential oil up to 72 h. After a 12 h exposure period the containers were opened and the dead beetles were removed and recorded. Knocked-down adults were regarded as alive if they showed continued movement of their appendages by the touch of a fine brush. After observation, the containers were tightly closed. After every 12 h, new batches of twenty insects were released and the numbers of dead insects were recorded and the percentage of mortality was calculated and corrected using Abbott's formula [27].

### 2.9. Data Analysis

Average percentage insect mortality was calculated from three replicates. Dosage-mortality responses were used to estimate LC_50_ (concentration causing 50% mortality) and LC_90_ (concentration causing 90% mortality) values at 24 and 48 h with Probit analysis [28] using Statgraphics 5.1 software. Insecticidal activity was considered significantly different when the 95% CI (confidence interval) fail to overlap.

## 3. Results

### 3.1. Chemical Composition of* Morinda lucida*

The chemical composition analysis by GC-MS identified 63 components of* M. lucida* essential oil, containing mainly 1,8-cineole (43.4%) followed by *α*-terpinyl acetate (14.5%), sabinene (8.2%), and *β*-pinene (4.0%; Table 1).

### 3.2. Mortality of* Callosobruchus maculatus*

**Figure 1:**
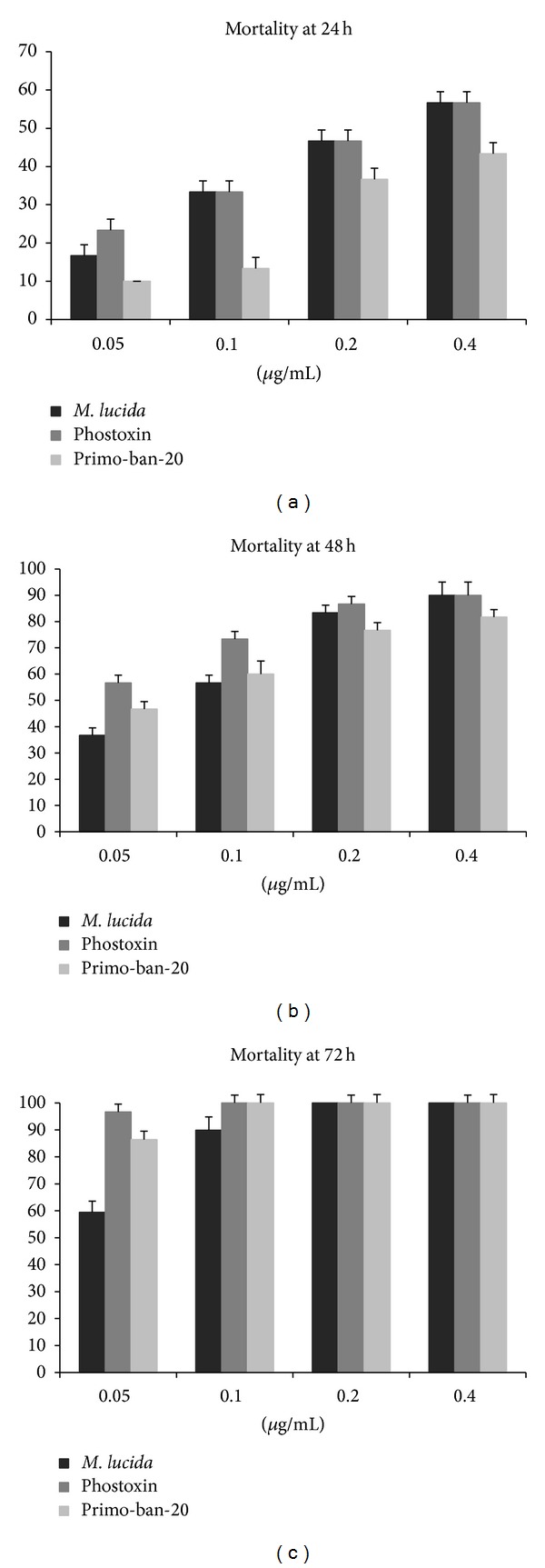
Effect of the* Morinda lucida* essential oil and pesticides against* Callosobruchus maculatus* at different concentrations and exposure times.

The aerial parts of the* M. lucida *distillate yielded a 0.48% (v/w) essential oil. The insecticidal bioassay of the essential oil of* M. lucida *against* C. maculatus *(Figure 1) showed that the activity of the oil was both dose dependent and exposure dependent. At a dose of 0.05 *μ*g/mL, the essential oil produced 59.4% mortality after 72 h (Figure 1(c)). The essential oil produced 33.3%, 56.7%, and 89.9% mortality after 24, 48, and 72 h at a dose of 0.10 *μ*g/mL, respectively, while a dose of 0.20 *μ*g/mL yielded mortality of 46.7%, 83.3%, and 100%, respectively, over the same time duration (Figure 1). The highest concentration of 0.40 *μ*g/mL produced a mortality of 90% and 100% after 48 and 72 h, respectively (Figures 1(b) and 1(c)).

The synthetic pesticide Primo-ban-20 at 0.20 *μ*g/mL had a lower mortality (36.7%) at 24 h, while Phostoxin had a 46.7% and 86.7% mortality after 24 h and 48 h at 0.20 *μ*g/mL, respectively (Figures 1(a) and 1(b)). Both* M. lucida* essential oil and pesticides reached 100% mortality at 72 h at 0.20 *μ*g/mL (Figure 1(c)). The negative control showed no activity except at 72 h (1.7%).

### 3.3. Determination of Insecticidal Activity

Probit analysis showed that* M. lucida* essential oil after 24 h presented an insecticidal activity (LC_90_ = 0.629 *μ*g/mL) similar to Phostoxin (LC_90_ = 0.652 *μ*g/mL) and Primo-ban-20 (LC_90_ = 0.726 *μ*g/mL). At 24 h essential oil of* M. lucida *(LC_50_ = 0.298 *μ*g/mL) and Phostoxin (LC_50_ = 0.295 *μ*g/mL) were more efficient than Primo-ban-20 (LC_50_ = 0.393 *μ*g/mL). At 48 h after exposure, there was no significant difference in LC_50_ between* M. lucida essential* oil compared to Primo-ban-20 and Phostoxin (Table 2, where all confidence intervals overlap).

## 4. Discussion

Recently, biological activity has been demonstrated for the* M. lucida* essential oil, such as the complete suppression of aflatoxin synthesis in maize [29], cytotoxic activity on cancer cell lines [30], and antioxidant and anti-inflammatory activity [20]. This study demonstrated a potent insecticidal activity for* M. lucida*, showing 100% mortality on* C. maculatus* at concentrations of 0.20 and 0.40 *μ*g/mL after 72 h (Figure 1).

Our results for* M. lucida* essential oil are consistent with other reports of essential oils that exhibited insecticidal and repellency activity against* C. maculatus, such as Eucalyptus intertexta, Callistemon viminalis, Ocimum americanum, Hyptis suaveolens, Hyptis spicigera, Lippia multiflora, Cymbopogon citratus, and Lippia rugosa *[25, 31–35]. Chaubey [36] evaluated the insecticidal, oviposition, egg hatching and developmental inhibitory activities of seven different essential oils against* Callosobruchus chinensis *and found that* Nigella sativa* was the most effective at all stages. Moreover, Kéita et al. [16] found that essential oils of* Ocimum basilicum* and* O. gratissimum* at a dose of 25 *μ*g/mL after 12 h of fumigation produced 80% and 70% mortality, respectively. Similarly, Moharramipour et al. [37] have shown a repellency of 82.4% of the* Thymus persicus* essential oil against* C. maculatus*.

The chemical components of this essential oil have been previously reported [38, 39], although there were differences in the ratio of their chemicals constituents. Okoh et al. [38] identified 50 compounds where *α*-terpinene was the major constituent with 17.8%, while in this study it represented only 0.8%. This difference could be attributed to the number of compounds identified in each study, since in this study, 63 components were identified, representing 95.4% of the total oil content. The essential oil was found to be dominated by oxygenated monoterpenes (Table 1).

Several essential oils components have shown insecticidal activity against* C. maculatus*. The leaf oil of* Cymbopogon schoenanthus, *rich in piperitone, gave 90% mortality after 24 h at a concentration of 6.7 *μ*L/L [40].* Plectranthus grandifolius* essential oil, predominantly (E)-myroxide, was shown to be toxic to both adults and eggs of* C. maculatus. *Likewise,* Cinnamomum aromaticum *bark oil, rich in* cis*-cinnamaldehyde, was insecticidal with an LC_50_ of 27.6 *μ*g/cm^2^ after 24 h [41]. The essential oil from* Cymbopogon giganteus*, rich in limonene and* p*-mentha-1(7),8-dien-2-ol, demonstrated insecticidal activity against* C. maculatus* and* C. subinnotatus* [42]. The concentrations of the major terpenoids constituents presented here for* M. lucida *essential oil are consistent with use of this plant as an insecticide and insect repellent.

The insecticidal activity of the* M. lucida* essential oil could be attributed to those known major components of oxygenated monoterpenes: 1,8-cineole (43.4%), *α*-terpinyl acetate (14.5%), 4-terpinen-4-ol (3.4%), *α*-terpinol (3.4%), and monoterpene hydrocarbons: sabinene (8.2%) and *β*-pinene (4.0%). These are compounds whose insecticide and repellent activities have been reported previously [32, 43, 44]. Nevertheless, it also has been shown that minor components may contribute to the biological activity [45] such as Myrcene, *α*-phellandrene, and Camphene.

The intensive use of synthetic insecticides not only produces genetic resistance by insect species but also causes serious problems of toxicity to humans and other animal species [6, 7]. Therefore, the essential oil extracted from* M. lucida* represents a botanical insecticide source, since it showed a potent insecticidal activity comparable to both pesticides, Phostoxin, and Primo-ban-20 (Table 2, where confidence intervals overlap). The lack of a statistical difference between* M. lucida* essential oil and two commercial pesticides is a positive result and demonstrates their equivalence, though we cannot conclude that* M. lucida* is more effective.

The results obtained indicate that* M. lucida* essential oil was biocidal even at lower concentration and may, therefore, be considered to be a useful alternative to synthetic insecticides.

## 5. Conclusions

This study demonstrated that essential oil extracted from* M. lucida* was toxic to pulse beetle* C. maculatus*. Therefore, it could be used as an alternative strategy for* C. maculatus *control and as a substitute for synthetic pesticides. However, further studies are necessary to elucidate the mode of action and their environmental impact and develop formulations to improve the insecticidal efficacy. This would benefit agricultural sectors of developing countries as these essential oils are readily available and biodegradable. The problem of volatility can be resolved through a controlled release formulation of their active chemical compositions.

## Figures and Tables

**Table 1:** Retention index and relative composition of major chemical components of *M. lucida* essential oil.

Component	KI^a^	%
*α*-Thujene	902	0.6
Tricyclene	908	Tr
Camphene	925	0.4
Sabinene∗	950	**8.2**
*β*-Pinene∗	954	**4**
Myrcene	966	0.9
*α*-phellandrene	1008	0.9
*δ*-3-Carene	1014	0.1
*α*-Terpinene	1021	0.8
*o*-Cymene	1027	Tr
*p*-Cymene∗	1029	1.2
Limonene∗	1034	2.4
1,8-Cineole∗	1037	**43.4**
(*E*) *β*-Ocimene	1054	Tr
g-Terpinene	1064	1.1
*Cis*-Sabinene hydrate	1073	0.5
Terpinolene	1091	0.4
Linalool∗	1103	2.2
*Cis*-*p*-Menth-2-en-1-ol	1127	0.3
*α*-Campholenal	1131	Tr
*Trans*-*p*-Menth-2-en-1-ol	1146	0.3
Camphor	1149	Tr
*β*-Pinene oxide	1162	Tr
Pinocarvone	1167	Tr
Borneol	1170	0.5
*δ*-Terpineol	1172	0.6
Terpinene-4-ol	1181	3.4
*p*-Cymen-8-ol	1188	Tr
*Trans*-*p*-Mentha-1(7),8-dien-2-ol	1191	Tr
*α*-Terpineol	1193	3.4
Myrtenal	1196	Tr
*Trans*-Piperitol	1211	Tr
*Trans*-Carveol	1223	Tr
Nerol	1228	0.2
Cuminaldehyde	1246	Tr
Carvone	1249	Tr
*Cis*-Verbenyl acetate	1283	0.1
Isobornyl acetate	1290	0.6
*Trans*-Sabinyl acetate	1292	0.1
*Trans*-Pinocarvyl acetate	1302	0.1
*Neo*-verbanol acetate	1321	0.8
*α*-Terpinyl acetate∗	1355	**14.5**
Eugenol	1364	0.6
*Cis*-Carvyl acetate	1367	Tr
Neryl acetate	1370	0.3
Geranyl acetate	1388	Tr
*β*-Elemene	1394	Tr
Methyl eugenol	1409	1.4
*β*-Caryophyllene	1421	Tr
*p*-Cymen-7-ol-acetate	1423	Tr
(*E*)-Cinnamyl acetate	1450	Tr
(*E*)*-*Methyl isoeugenol	1495	Tr
Bicyclogermacrene	1497	Tr
*α*-Bulnesene	1506	Tr
*β*-Sesquiphellandrene	1526	Tr
Elemol	1554	Tr
Elemicin	1557	Tr
Spathulenol	1578	0.4
Caryophyllene oxide	1583	0.5
g-Eudesmol	1635	Tr
*β*-Eudesmol	1651	0.2
*α*-Eudesmol	1655	Tr
Intermedeol	1667	Tr
Total identified		**95.40**

Major components are shown in bold.

*These chemical components were identified by authentic compound injection.

^a^KI Kovats retention indices.

Tr = trace amount <0.1%.

**Table 2:** Insecticidal activity of *M. lucida* essential oil against *C. maculatus*.

Test sample	24 h exposure			48 h exposure		
	LC_50_	LC_90_	*χ* ^2a^	LC_50_	LC_90_	*χ* ^2a^
*M. lucida* essential oil	0.298	0.629	18.3	0.122	0.308	36.4
	(0.251–0.368)	(0.521–0.819)		(0.098–0.147)	(0.265–0.372)	
Phostoxin	0.295	0.652	17.9	0.081	0.286	58.6
	(0.245–0.370)	(0.532–0.870)		(0.052–0.106)	(0.241–0.357)	
Primo-ban-20	0.393	0.726	12.1	0.126	0.387	41.5
	(0.331–0.495)	(0.596–0.965)		(0.094–0.159)	(0.326–0.484)	

Units LC_50_ (concentration causing 50% mortality) and LC_90_ (concentration causing 90% mortality) in units of *μ*g/mL. 95% confidence interval (CI) is shown in parenthesis. Insecticidal activity is considered significantly different when the 95% CI fail to overlap. ^a^Chi-square value of Probit model fit, all significant at *P* < 0.05 level.
